# Sr^2+^ sorption property of seaweed-like sodium titanate mats: effects of crystallographic properties†

**DOI:** 10.1039/d1ra03088d

**Published:** 2021-05-24

**Authors:** Yoshifumi Kondo, Tomoyo Goto, Tohru Sekino

**Affiliations:** The Institute of Scientific and Industrial Research (ISIR-SANKEN), Osaka University 8-1 Mihogaoka Ibaraki Osaka 567-0047 Japan goto@sanken.osaka-u.ac.jp sekino@sanken.osaka-u.ac.jp +81-6-6879-8439 +81-6-6879-8436; Division of Materials and Manufacturing Science, Graduate School of Engineering, Osaka University 2-1 Yamadaoka Suita Osaka 565-0871 Japan; Institute for Advanced Co-Creation Studies, Osaka University 1-1 Yamadaoka Suita Osaka 565-0871 Japan

## Abstract

Layered sodium titanate is a typical ion-exchanger for water purification aimed at removing cationic heavy metals and radionuclides. The material design of an ion-exchanger is effective for cation removal. For that purpose, understanding the basic impacts of crystallographic properties such as crystal size, morphology, and phase is critical for developing highly functional nanoscale ion-exchangers. In this study, we investigate the principal relationship between the crystallographic properties of seaweed-like sodium titanate mats (SSTs), which consist of a dititanate (H_*x*_Na_2−*x*_Ti_2_O_5_) phase of nanofibers synthesised by the alkaline hydrothermal method and their Sr^2+^ sorption mechanism. A trititanate (H_*x*_Na_2−*x*_Ti_3_O_7_) phase, which has a micro-sized fibre morphology, was also synthesised using the same method by adjusting the NaOH concentration. The SST demonstrates a high ion-exchange selectivity of Sr^2+^ against H^+^ and a high maximum sorption capacity (2 mmol g^−1^), which was four times higher than that of the trititanate phase (0.49 mmol g^−1^). In contrast, the trititanate phase, which is the comparison target, had a low Sr^2+^ ion-exchange selectivity and precipitated SrCO_3_. We conclude that these differences in Sr^2+^ sorption mechanisms were derived from not only the unique morphology but also the crystal structure of sodium titanates. Although almost all of the Na^+^ in dititanate with lamellar structure was consumed by the ion-exchange reaction, some Na^+^ remained in the trititanate because there are two sites in the zigzag layered structure. These findings on the crystallographic properties of SST for Sr^2+^ sorption may contribute to the functionalisation of a nanoscale ion-exchanger.

## Abstract 2

Seaweed-like sodium titanate mats (SSTs) have excellent sorption capacity of Sr^2+^ compared to sodium trititanate monodispersed nanofibers due to the sodium dititanate structure and its unique nano/micro-scale morphologies.

## Graphical abstract

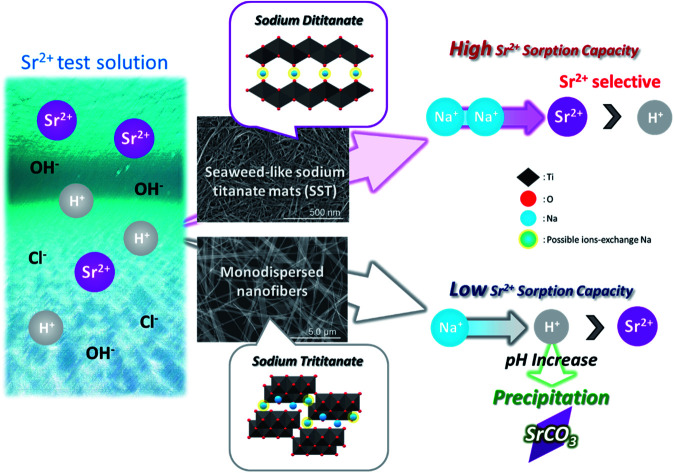

## Introduction

The demand for energy has reached an unprecedented level due to rapid population growth and dramatic global economic growth.^1–3^ The use of nuclear power, which generates large amounts of energy with low greenhouse gas emission, has been proposed as a solution.^3,4^ However, nuclear power generation poses the problem of the treatment of wastewater contaminated by radionuclides (^137^Cs, ^90^Sr, *etc.*).^5,6^ These radionuclides are harmful to human health and the environment, and must be removed from wastewater.^7–9^ In particular, ^90^Sr is known to be one of the most dangerous radioactive species because it has a long half-life of 28.9 years;^10–12^ it mainly accumulates in bone tissue.^12^ Radioactive ^90^Sr is not common in nature, but stable isotopes of strontium are commonly found in soil and groundwater. In 2014, The United States Environmental Protection Agency declared the preliminary regulatory determination of strontium at 1500 μg L^−1^ in drinking water as a health reference level.^13^

Numerous efforts have been undertaken to develop purification methods that can remove strontium from water effectively. For instance, purification materials used for water purification include layered titanate,^14–17^ zeolite,^18,19^ hydroxyapatite,^20–22^ organic materials,^23,24^ and others. Among these materials, sodium titanate is established as an efficient strontium removal material. Commercially available sodium titanate-based material for radioactive strontium removal has been used for the treatment of wastewater at the Fukushima Daiichi Nuclear Power plant (FDNP), and its sorption capacity of Sr^2+^ was reported as approximately 1.4 mmol g^−1^ in simulated seawater.^25^ For FDNP, radionuclides including Sr^2+^ were removed by a multi-step process *via* several types of adsorbents. Although it is possible to enhance the purification properties by designing a multi-step process, it is also important to enhance the removal efficiency of the individual materials by improving the characteristics of the adsorbent carrier. Sodium titanate nanostructures are easily produced *via* alkaline hydrothermal synthesis,^26–28^ and they have a layered structure, with TiO_6_ octahedra in the host layer and sodium ions (Na^+^) in the guest layer. The Na^+^ ions in sodium titanate are able to undergo cation exchange with polluting species in wastewater. Moreover, sodium titanate is known to exhibit various chemical formulas and crystal structures,^29,30^ and its ion-exchange properties have been reported so far.^31–33^ However, only a few reports have discussed the relationship between the sorption behaviour of cations and crystallographic properties from the perspective of ion-exchange effectiveness.^31,32,34^ The removal efficiency of sodium titanates for several cations, including heavy metals and radionuclides, has been reported.^9,14–17^ Recently, the Sr^2+^ adsorption selectivity of sodium nonatitanate (Na_4_Ti_9_O_20_) and sodium trititanate (Na_2_Ti_3_O_7_) were investigated by Kunishi *et al.*^35^ Although several references have reported on the mechanism of sodium titanate sorption, it is interesting to note that the ion-exchange reaction has been the focus for the cation removal by sodium titanates, while other reactions, such as precipitation or surface adsorption, have been overlooked. Recently, we reported the synthesis of a unique seaweed-like sodium titanate mat (SST), which is composed of randomly distributed layered-sodium titanate nanofibers, using a template-free alkaline hydrothermal process.^36^ The SST showed a high Co^2+^ sorption capacity by ion-exchange reactions without self-precipitation of cobalt hydroxide in comparison with commercially available Na_2_Ti_3_O_7_. Although this previous study underscores the importance of understanding the reaction mechanism of cation removal on the functionalisation of sorbents, the synthesis conditions, such as the effect of the alkaline concentration on crystallographic properties of the SST, have not been sufficiently discussed.^36^ Alkaline concentration (Na^+^) and solution pH are important factors for the formation of sodium titanate,^26^ and therefore, optimisation of the material structure synthesis process is indispensable for improving its properties.

Herein, we propose an effective optimal-synthesis condition of an SST mat with a low-crystalline and high surface area and permeability for water purification. The sodium titanates, including the SST, were synthesised by the alkaline hydrothermal process using various Na^+^ concentrations, and we investigated its sorption behaviour for Sr^2+^ from test solutions. We selected Sr^2+^, which is a radionuclide regarded important in terms of application, as target cation for this investigation. In addition to investigating the effects of synthesis conditions on crystallographic properties, such as the phase, morphology, and composition, of sodium titanates by the hydrothermal method, we discuss the relationships between the detailed crystallographic characterisation of the SST and the sorption capacity.

## Experimental

### Synthesis of sodium titanates by the alkaline hydrothermal method

Sodium titanate samples were prepared by an alkaline hydrothermal synthesis. Titanium sulfate solution (Ti(SO_4_)_2_) (6.87 mL) and ultra-pure water (9.13 mL) were added to a sodium hydroxide (NaOH) aqueous solution (40 mL) with stirring. The mixture was transferred to a 100 mL Teflon-liner in a stainless-steel autoclave (SAN-AI Kagaku Co. Ltd, Aichi, Japan) and heated at 200 °C for two days. The products were filtered and washed with ultra-pure water several times. The obtained white solid was freeze-dried for two days. A series of T*X*M samples (“*X*” indicates the concentration of the NaOH aqueous solution used) were obtained using NaOH concentrations of 1.0, 5.0, 10, and 15 M in the hydrothermal synthesis. Material information and additional experimental procedure are described in the ESI.†

### Characterisation

The synthesised materials before and after sorption test were characterised by the following methods. Powder X-ray diffraction (XRD) patterns were recorded using a D8 ADVANCE (Bruker AXS GmbH, Karlsruhe, Germany) with Cu Kα radiation (*λ* = 1.5418 Å) at 40 kV and 40 mA. The Ti K-edge and Sr K-edge of X-ray adsorption fine structure (XAFS) were measured using an ionisation chamber in transmission mode on the Kyushu University Beamline (BL-06) of the Kyushu Synchrotron Light Research Centre (SAGA-LS; Tosu, Japan). Pellet samples for Ti K-edge XAFS measurements were prepared as mixtures with boron nitride. In the case of Sr K-edge, pellet samples were prepared without dilution, except for the reference sample. Ti K-edge and Sr K-edge XAFS spectra were collected over a photon energy range of 4635.2–5938.6 eV and 15 778.1–16 844.3 eV, respectively. The collected spectra were analysed using the REX2000 software (Rigaku Co., Tokyo, Japan). TiO_2_ (anatase), strontium chloride (SrCl_2_), and strontium carbonate (SrCO_3_) were used as reference samples for XAFS analysis. The specific surface area (*S*_BET_) was determined by nitrogen (N_2_) adsorption isotherm data collected at −196 °C using a NOVA4200e (Quantachrome Instruments, FL, USA), and was measured by the multi-point Brunauer–Emmett–Teller (BET) method (0.05 < *p*/*p*_0_ < 0.30). Field emission-scanning electron microscopy (FE-SEM) images were obtained using an SU9000 electron microscope (Hitachi High-Technologies Co., Tokyo, Japan) at 20 kV. Transmission electron microscopy (TEM) images were obtained using a JEM-2100 electron microscope and JEM-ARM 200F (JEOL Ltd., Tokyo, Japan). Elemental analysis of the sorbent was also performed through SEM equipped with energy-dispersive X-ray spectroscopy (EDS, X-MaxN100LE, HORIBA, Ltd., Kyoto, Japan). X-ray photoelectron spectroscopy (XPS) measurements were carried out on a JPS-9010MX (JEOL Ltd., Tokyo, Japan) photoelectron spectrometer, using Mg Kα X-ray irradiation (1253.6 eV). The binding energy was calibrated with the adventitious carbon (C 1s) peak at 285.0 eV. The solution pH after the sorption test was measured using a pH meter (D-52, HORIBA, Ltd., Kyoto, Japan). The zeta potential was measured using the electrophoretic method on an analyser (Zetasizer Nano ZS, Malvern Instruments Ltd, Malvern, UK).

### Sr^2+^ sorption test

The Sr^2+^ test solution, between 0.2 mM and 4.0 mM, was prepared using SrCl_2_ without pH adjustment (pH = 4–5) to investigate the simple ion-exchange reaction without a buffer. It is known that pH affects adsorption, and that maximal adsorption has been observed over a wide pH range of 4–11.^15^ For the sorption test, as-synthesised T1M, T5M, T10M, and T15M were used. Sorbents (10.0 mg) were placed in plastic tubes, and 20 mL of SrCl_2_ solutions at various concentrations were added to them. The tubes were shaken at a constant speed of 150 rpm in a mechanical shaker (Bio Shaker, BR-43FL, TAITEC CORPORATION, Saitama, Japan) at 25 °C for one day. Subsequently, the sorbent and solution were separated by filtration, and the sorbents were freeze-dried to obtain powder samples. The characterisation of the samples after the sorption test is described in the ESI.† The sorption isotherm and removal efficiency of Sr^2+^ were evaluated from the results of elemental analysis by inductively coupled plasma optical emission spectrometry (ICP-OES; Optima 8300, PerkinElmer Japan Co., Ltd., Yokohama, Japan). Detailed analysis methods are described in the ESI.†

### Evaluation of sorption characteristics

The Sr and Na concentrations of the solution after sorption testing were analysed by ICP-OES. The sorption results of Sr were fitted using the Langmuir equation:1
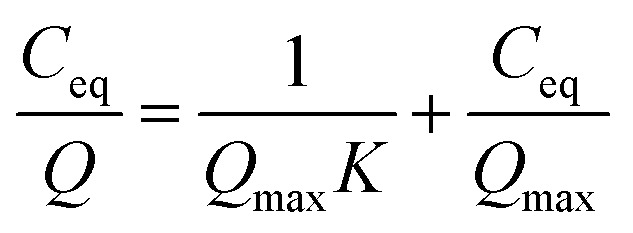
where *Q* is the amount of adsorbed Sr per gram (mmol g^−1^), *C*_eq_ is the equilibrium concentration of Sr (mmol L^−1^), *Q*_max_ is the maximum adsorbed Sr (mmol g^−1^), and *K* is a constant related to the adsorption rate coefficient. The sorption isotherm of Sr was also fitted to the Freundlich equation:

2 *Q* = *K*_F_*C*_eq_^1/*n*^

where *Q* is the amount of adsorbed Sr per gram (mmol g^−1^), *C*_eq_ is the equilibrium concentration of Sr (mmol L^−1^), and *K*_F_ ((mmol g^−1^) (L mmol^−1^)^1/*n*^) and *n* are the Freundlich constants corresponding to adsorption capacity and adsorption intensity. As in other reports, by assuming that the ion-exchange reaction can be approximated as the surface adsorption reaction, in this study, the sorption isotherm was investigated using the Langmuir and Freundlich models. The removal efficiency (%) of Sr from test water was calculated using the following equation:3
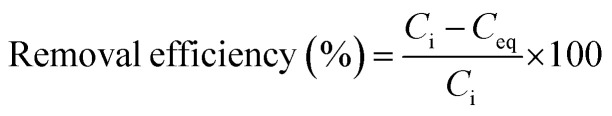
where *C*_i_ and *C*_eq_ are the initial concentration of Sr (mmol L^−1^) and the equilibrium concentration of Sr in the test solution (mmol L^−1^), respectively. The ion-exchange capacity (IEC, mmol g^−1^) of the sample was calculated using the following equation:4
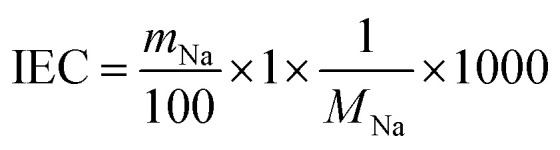
where *m*_Na_ is the Na concentration (wt%) of the sample, determined from ICP-OES analysis, and *M*_Na_ (g mol^−1^) is the atomic weight of Na.

## Results & discussion

### Characterisation of sodium titanate samples

**Fig. 1:**
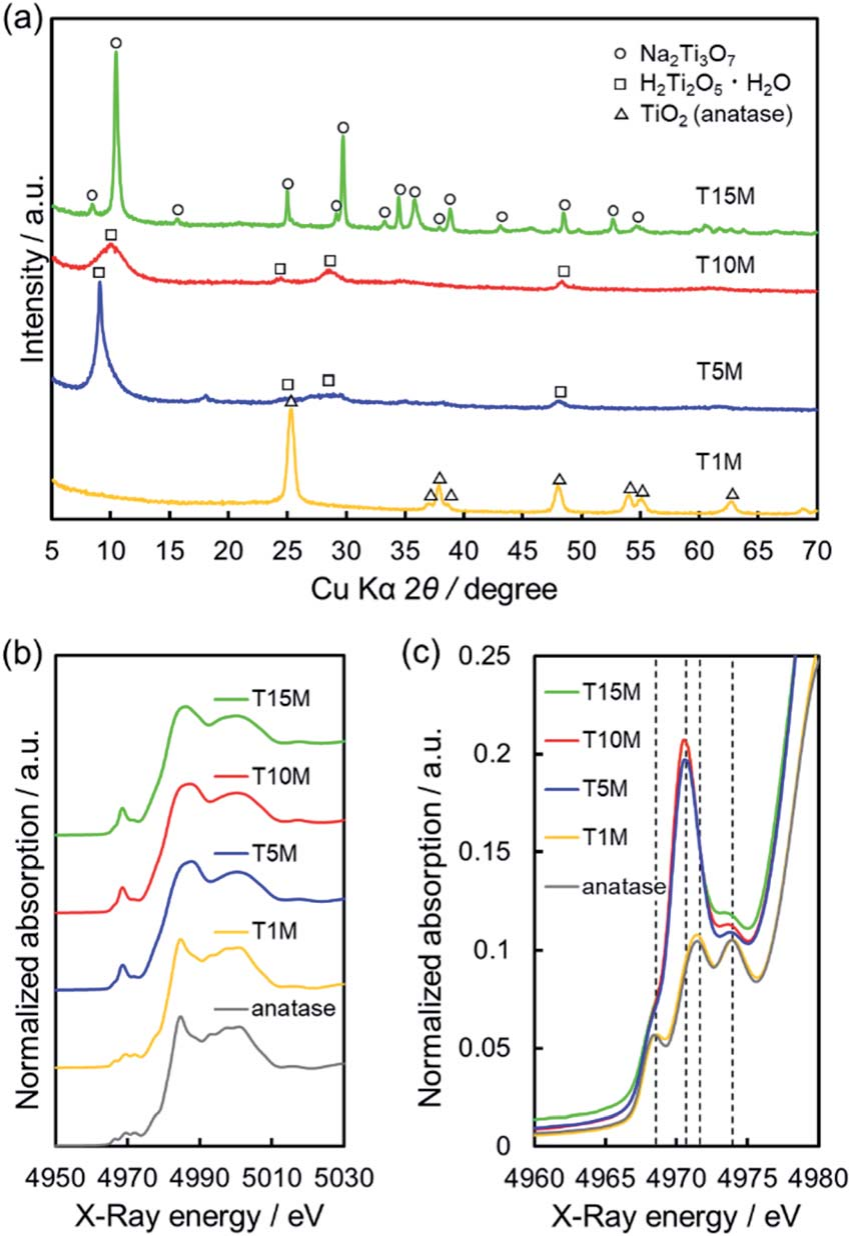
(a) XRD patterns, (b) Ti K-edge XANES spectra, and (c) pre-edge peak of Ti K-edge XANES spectra of anatase (black), T1M (yellow), T5M (blue), T10M (red), and T15M (green).

Sodium titanate (T*X*M) samples, including the SST, were synthesised by the alkaline hydrothermal method with various NaOH concentrations. Fig. 1a shows the powder XRD patterns of T1M, T5M, T10M, and T15M, respectively. T1M was indexed to the anatase phase of TiO_2_ (powder diffraction files (PDF) no. 01-086-1157). T5M and T10M were identified as layered hydrated dititanate (H_2_Ti_2_O_5_·H_2_O) (PDF no. 00-047-0124).^26,27,37^ T10M had the same synthesis condition as the SST.^36^ T15M corresponded to the crystal planes of layered sodium trititanate (Na_2_Ti_3_O_7_) (PDF no. 01-072-0148).^14,28,31^ The *d* values at 200 reflection for T5M and T10M, and at 100 reflection for T15M were estimated to be 9.87, 8.88, and 8.40 nm, respectively, calculated by the Bragg equation. The interlayer spacing of the dititanate samples (T5M and T10M) was wider than that of the trititanate sample (T15M). The interlayer spacing of sodium titanate may decrease due to the difference in crystal structure from orthorhombic dititanate to monoclinic trititanate determined by the Na concentration during hydrothermal synthesis.^26^

**Fig. 2:**
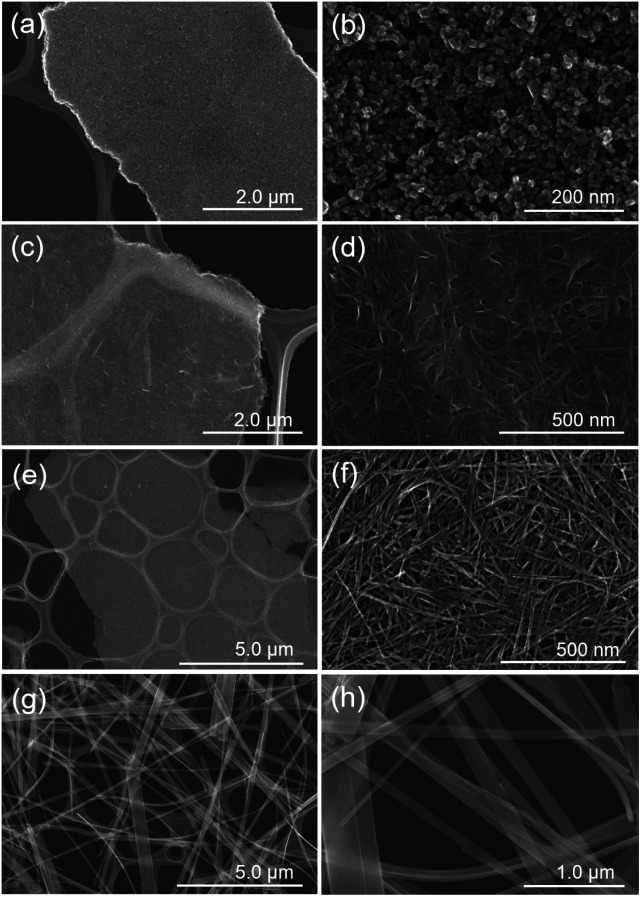
FE-SEM images of (a and b) T1M, (c and d) T5M, (e and f) T10M, and (g and h) T15M.

With reference to the above XRD results, the chemical composition of Na and Ti was determined using ICP-OES analysis. The chemical composition of T1M, T5M, T10M, and T15M is shown in Table 1. The Na amount of the titanates increased with increasing Na concentrations in the hydrothermal synthesis, and all samples were estimated to contain H^+^.^38^ XAFS measurements were performed to investigate the effect of the hydrothermal alkaline condition on the local structure of the T*X*M samples. Fig. 1b shows the Ti K-edge X-ray absorption near-edge structure (XANES) spectra of the T*X*M and reference samples. The Ti K-edge XANES spectrum of T1M was consistent with that of anatase, similar to the result shown by the XRD analysis. Fig. 1c shows that the pre-edge peaks of T5M, T10M, and T15M were characteristic of five-coordinate titanium.^39,40^ This is because the TiO_6_ octahedra of sodium titanate share edges.^14^ In addition, T15M had a local structure similar to that of the Na_2_Ti_3_O_7_ reference sample in the Ti K-edge Fourier transformed extended X-ray absorption fine structure (FT-EXAFS) spectra of the T*X*M samples (Fig. S1†). These XAFS results were in good agreement with the XRD results. The structure and morphologies of the titanate samples were investigated by FE-SEM. The FE-SEM images of T1M, T5M, and T10M showed a seaweed-like secondary structure at the microscale (Fig. 2a, c and e). Through the observation at the nanoscale, T1M was found to be made up of nanoparticles (14.6 ± 3.14 nm) on thin-plates.

**Table 1:** Elemental weight ratio and estimated chemical composition of T*X*M samples

Sample	Na (wt%)	Ti (wt%)	Chemical composition	*S*_BET_ a (m^2^ g^−1^)
T1M	0.38	51.1	TiO_2_	94.7
T5M	7.52	43.1	H_1.27_Na_0.73_Ti_2_O_5_	90.6
T10M	8.34	38.8	H_1.10_Na_0.90_Ti_2_O_5_	167.8
T15M	12.7	44.8	H_0.23_Na_1.77_Ti_3_O_7_	12.2

a Determined by the multi-BET method by N_2_ adsorption; data ranging from *p*/*p*_0_ = 0.05 to 0.30.

In the case of T5M samples, unclear nanoplates and nanofibers of various shapes and sizes were observed on thin-plates. In contrast, T10M was made up of thin-plates comprising nanofibers (width: 8.68 ± 0.94 nm, length: 289 ± 66 nm). The macropore structure implies that disordered aggregation occurred *via* nanoplates or nanofibers. In contrast, T15M was made up of monodispersed long microfibers (width: 146.8 ± 103.2 nm, length: 77.0 ± 22.7 μm). These differences in the shape and size of the components of T5M and T10M caused the differences in the macropore structures, as also indicated by the results of the N_2_ adsorption–desorption isotherms (Fig. S2†). As shown in Fig. S2,† the isotherms of T5M and T10M exhibited a type II behaviour and H3 type hysteresis loop, which is consistent with the fact that these samples comprised thin-plate-like aggregates. T1M, the isotherm of which was type V, was estimated to the aggregates. In addition, T15M exhibited a type III isotherm, reflecting the absence of macropores. The specific surface area (*S*_BET_) of T10M was 168 m^2^ g^−1^, which was higher than that of any other titanate (Table 1).

**Fig. 3:**
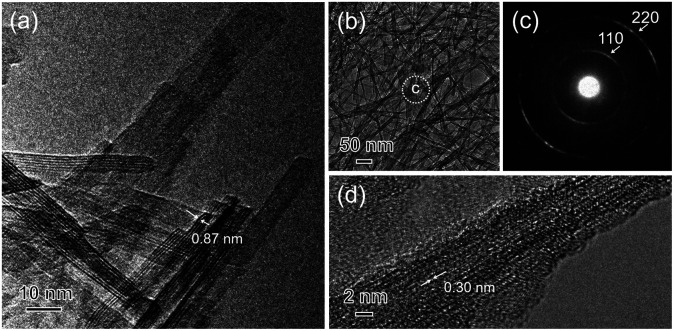
(a and b) TEM image, (c) SAED pattern of the analysis area of (b), and (d) HRTEM images of the T10M (SST) sample.

Furthermore, to examine the layered structure of the samples, the primary nanostructures of T5M, T10M, and T15M were observed by TEM. Fig. 3 and S3† show TEM images, selected area electron diffraction (SAED) patterns, and high-resolution TEM (HRTEM) images of the titanate samples. A layered structure was clearly observed in their fibres. In the case of T10M, fibre crystals of uniform size were intertwined (Fig. 3a and b), and ring diffraction patterns were observed due to the presence of nanocrystals in various orientations (Fig. 3c). Based on the different observation areas, the measured fringe spacing was approximately 0.30 nm, which was estimated to correspond to a *d* value of (600) for the data for H_2_Ti_2_O_5_·H_2_O (PDF no. 00-047-0124) (Fig. 3d). An aggregate containing large plate-like and amorphous crystals was observed in T5M (Fig. S3a and b†), and the SAED pattern of T5M was consistent with the data for H_2_Ti_2_O_5_·H_2_O (PDF no. 00-047-0124) (Fig. S3c†). The HRTEM image indicated that T5M had a high crystallinity because a lattice structure could be clearly observed (Fig. S3d†). In contrast, long plate-like crystals were observed in T15M (Fig. S3e and f†), and the SAED patterns identified these plate-like crystals as a Na_2_Ti_3_O_7_ phase (PDF no. 01-072-0148) (Fig. S3g†). From the observations of different crystal locations, a layer structure could be clearly observed (Fig. S3h†). The interlayer distances of T5M, T10M, and T15M, observed by TEM, were approximately 0.97, 0.87, and 0.83 nm, respectively. These results were in good agreement with the calculations using the Bragg equation and the XRD results.

### Sr^2+^ sorption capacity

**Fig. 4:**
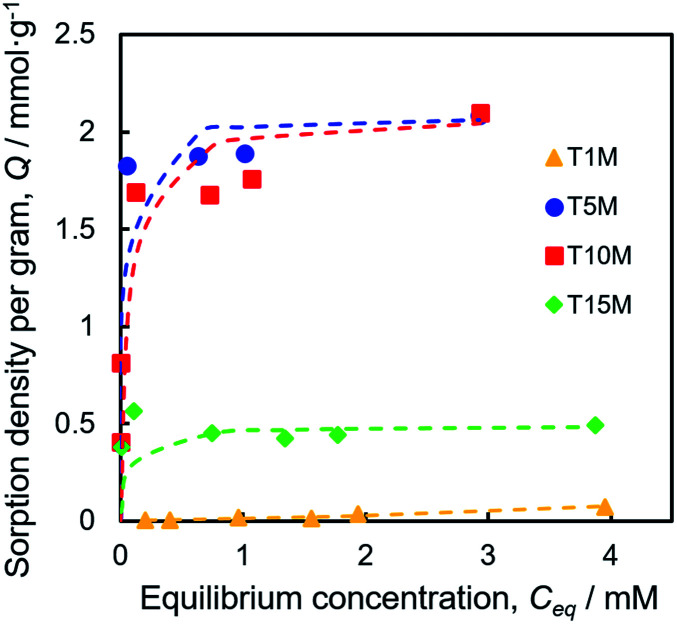
Sr^2+^ sorption isotherms fitted with the Langmuir plot of T*X*M samples.

To investigate the Sr^2+^ sorption behaviour, batch tests were performed by shaking at 25 °C for one day, using the 0.2–4.0 mM SrCl_2_ aqueous solution. Fig. 4 demonstrates that the Sr^2+^ sorption isotherms of T*X*M were better fitted to the Langmuir equation as compared to the Freundlich model fitting, as shown in Fig. S4a and Table S1.† The Sr^2+^ sorption of T1M was below the detection limit (Fig. 4). From the Langmuir model, the maximum sorption density per gram (*Q*_max_) was calculated to be 2.08, 2.09, and 0.49 mmol g^−1^ for T5M, T10M, and T15M, respectively. That is, the *Q*_max_ was smaller in the order T10M ≈ T5M > T15M > T1M, and the dititanate phase samples (T5M and T10M) had a much higher Sr^2+^ sorption capacity than the trititanate phase (T15M). The Sr^2+^ removal efficiencies of the samples against the various initial concentrations of the SrCl_2_ solution are presented in Fig. S4b.† T5M and T10M had a removal efficiency of 80% or more, with initial concentrations under 1 mM. This indicated that the dititanate phase has a good performance in the low concentration range. The theoretical ion-exchange capacity (IEC) of the samples was calculated from eqn (4) using their Na concentrations (Table 2). The IEC is an index representing the amount of the ion-exchange reaction. The IEC increased in the order T5M < T10M < T15M. The *Q*_max_ of T5M and T10M exceeded their IEC/2, perhaps, because the Sr^2+^ adsorbed on the surface of the titanates resulted in a negative surface charge and large specific surface area. Although the IEC/2 of the T15M sample was highest among the titanate samples, its *Q*_max_ was, interestingly, lowest, approximately 18% of its IEC/2.

**Table 2:** *Q*_max_ and theoretical ion-exchange capacity (IEC) of T5M, T10M, and T15M

Sample	*Q*_max_ a (mmol g^−1^)	IEC^b^ (mmol g^−1^)
T5M	2.08	3.27
T10M	2.09	3.63
T15M	0.49	5.52

a Calculated by Langmuir equation.

b Calculated from Na concentration of ICP-OES analysis. Assuming that Sr^2+^ exchanges with 2Na^+^, IEC/2 can be regarded as the adsorbable amount of Sr.

### Sr^2+^ sorption behaviour of sodium titanates

**Fig. 5:**
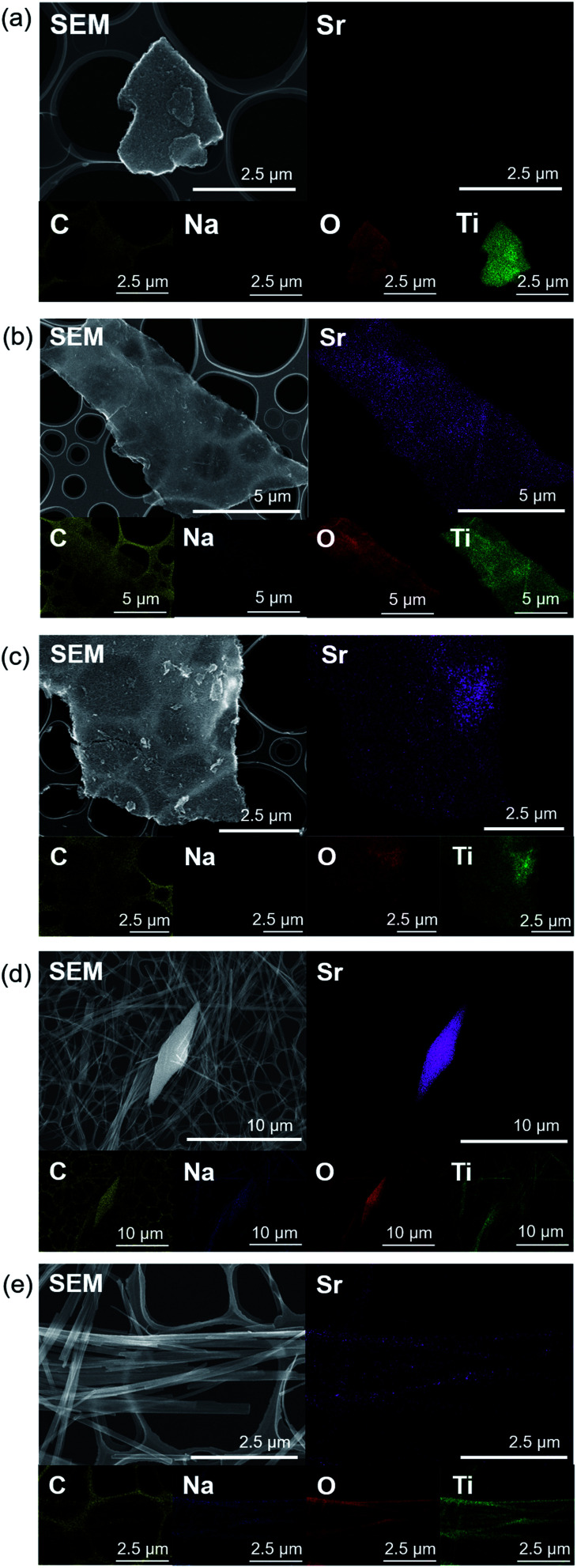
SEM-EDS mappings of (a) T1M, (b) T5M, (c) T10M, and (d and e) T15M after the sorption test using 4 mM SrCl_2_ solution.

**Fig. 6:**
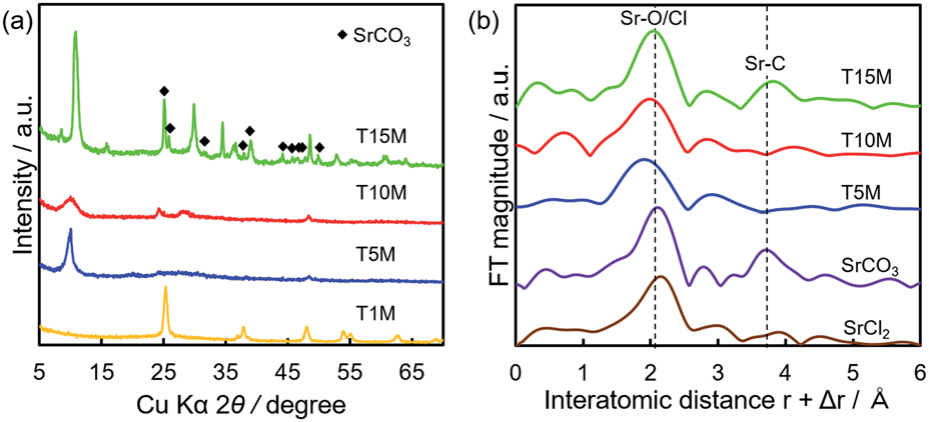
(a) XRD patterns and (b) Sr K-edge FT-EXAFS spectra of T*X*M after sorption tests using 4 mM SrCl_2_ solution.

Fig. 5 shows the SEM-EDS mappings of T*X*M samples after the sorption test. As shown in Fig. 5a, EDS mappings indicates that Sr was present in low amounts in T1M, as also reflected in the result of the Sr sorption isotherm (Fig. 4). In the case of T5M and T10M, no morphology changes or precipitates were observed after the sorption test. The Sr image overlapped with the Ti and O images on the EDS-mappings, and Na was present at the detection limit in T5M and T10M (Fig. 5b and c). The morphology of the layered titanate microfibers remained in T15M, and rhombus-like precipitates were also observed after the sorption test (Fig. 5d). This rhombus-like precipitate overlapped with Sr, C, and O images on the EDS-mappings. In addition, Sr in T15M was estimated to be intercalated into the interlayer or adsorbed on titanates, as indicated by the SEM-EDS mapping of T15M (Fig. 5e), because the Sr image was observed overlapping with the titanate microfibers. In addition, Na was detected in the same place as Sr, O, and Ti by the enlarged SEM-EDS (Fig. 5d and e) and by XPS (Fig. S5†). That is, a part of Na^+^ remained in T15M after the sorption test. Fig. 6 shows the XRD patterns and FT-EXAFS spectra of the samples after the sorption tests. The XRD patterns of T5M and T10M did not show peaks of Sr compounds after the sorption tests. However, the 200-diffraction peak of T5M was slightly shifted toward a high angle, as shown in Table S2 and Fig. S6c,† and the peak intensity of T10M was decreased and broadened (Fig. S6b†). The Sr K-edge FT-EXAFS spectra of T5M and T10M indicated that Sr species existed in the ionic state because the Sr–O peak of the first coordination shell was observed without secondary peaks (Fig. 6b). In other words, the analysis indicated that Sr^2+^ might be intercalated into the layered structures of dititanate, *i.e.*, T5M and T10M, as hydrated ions. Moreover, T15M, *i.e.*, the trititanate phase, showed new diffraction peaks derived from SrCO_3_ (PDF no. 00-005-0418), appearing along with the trititanate phase after the sorption test (Fig. 6a). In addition, the 100-diffraction peak of T15M was shifted toward a high angle, which implied the intercalation of Sr^2+^ or H^+^ into the interlayer (Table S2†). The FT-EXAFS spectrum of T15M after the sorption test showed two peaks, which could be assigned to the Sr–O and Sr–C coordination shells by comparison with the SrCO_3_ sample.

Thus, T15M exhibited the formation of SrCO_3_ (Fig. 6b). The results suggest that Sr^2+^ was removed from the solution by both self-precipitation and intercalation into the interlayer space of T15M by the ion-exchange reaction with Na^+^.^14,17^

### Relation between the amount of the released Na^+^ and final pH

During the ion-exchange reaction of sodium titanates, the solution pH increases with increasing OH^−^ concentration because of the ion-exchange between Na^+^ and H^+^, as shown in the following equation:X–Na + H_2_O ↔ X–H + Na^+^ + OH^−^,where X is one of the sodium titanate compounds. In other words, the pH change is indicative of the ion-exchange reactions with H^+^.^9^

**Fig. 7:**
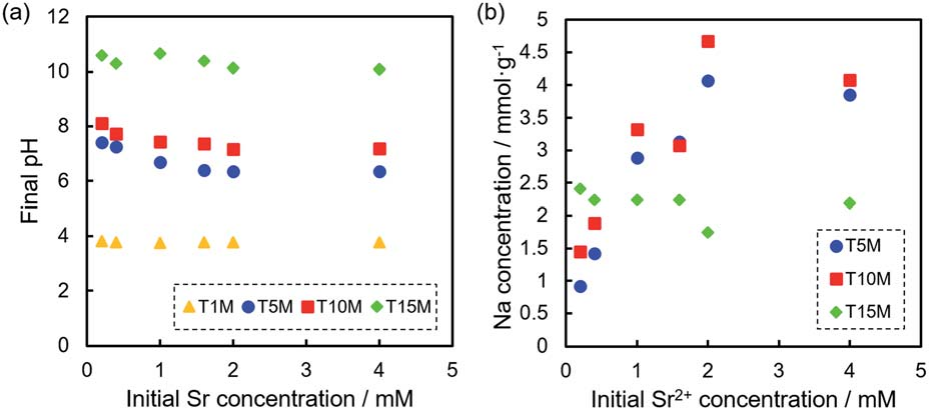
(a) pH of the solution after Sr^2+^ sorption tests at each Sr^2+^ concentration, and (b) Na concentration of the solution after sorption tests of T5M, T10M, and T15M.

The solution pH after the sorption tests was in the order of T15M > T10M ≥ T5M > T1M, and the solution pH of the samples increased from the initial pH (approximately 5.8) by the sorption test, except for T1M (Fig. 7a). For T15M, the solution pH after the sorption tests was approximately 10.5. SrCO_3_ might be easily precipitated on T15M by reaction with CO_2_ in air under shaking because SrCO_3_ easily precipitates in an alkaline environment (>pH 10).^41,42^ This explanation is also supported by the formation of rhombus-like precipitates identified as SrCO_3_ in the T15M sample. Fig. 7b shows the amount of Na^+^ released from the samples after the sorption test. The released Na^+^ concentration from T5M and T10M increased with increasing initial Sr^2+^ concentration. In contrast, the released Na^+^ concentration from T15M was almost constant, regardless of the initial Sr^2+^ concentration of the test solution. The pH increase in T15M was particularly high and might be attributed to the fact that Na^+^ in the interlayer was exchanged with H^+^.

From the results of the Sr sorption test, the concentration of the subtracted sorption of [Sr] from the released [Na]/2 was calculated to investigate the ion-exchange with Sr^2+^ and Na^+^, as shown in Fig. S7.†

At a low initial Sr concentration, the values of sodium dititanate (T5M and T10M) were approximately 0 mmol g^−1^, indicating the ion-exchange reaction between Sr^2+^ and Na^+^. On the other hand, in the case of T15M, the values were approximately −0.6 mmol g^−1^, regardless of the initial concentration, indicating that the released Na^+^ from T15M was higher than the removed Sr^2+^.

The possibility of an ion-exchange between Sr^2+^ and H^+^ in the interlayer was also inferred, because T15M contained H^+^, as shown in Table 1. To investigate this reaction, protonated titanates (T10M_HCl and T15M_HCl), which were synthesised by washing with HCl, were prepared and their ion-exchange reactions investigated. The results showed that the exchange of H^+^ with Sr^2+^ in titanates was difficult under our experimental conditions, despite no significant changes in the crystal phase by protonation (Fig. S8†).

Fig. S9† shows the changes in the zeta potential *versus* the solution pH. Except for T1M, all samples indicated a negative charge in the pH range of 2–10, and the zeta potential increased under acidic condition. This indicates that T5M, T10M, and T15M showed a negative charge during the adsorption test. As known from previous experiments,^36^ this fact shows that sodium titanates have an advantage in cation adsorption, indicating that cations that contain Sr^2+^ easily adsorb on the surface. However, the pH change may affect the zeta potential as well as the ion-exchange selectivity of the sorbents. This is because the ion-exchange with H^+^, shown in Fig. S8,† is expected to be significantly affected by the solution pH. In other words, these results indicate that the pH of wastewater has a significant effect on the sorption behaviour of sorbents.

### Relation between titanate crystal structure and Sr^2+^ sorption

As the basic reaction principle in the past, the sorption mechanisms of ion-exchange reactions with cations and Na^+^/H^+^ in titanate were well demonstrated, and the influencing factors on the mechanism have been discussed.^14,17,31,32,34^ However, despite numerous reports on the relationship between ion-exchange reactions and crystal structure, effects of differences in the crystal structure depending on the composition of sodium titanate and on the sorption mechanisms, including ion-exchange, adsorption, and precipitation, are still unclear. To investigate the optimal synthesis condition of the SST, we organised the effects of the crystallographic properties of sodium titanates on the sorption properties of Sr^2+^ as shown below.

**Scheme 1:**
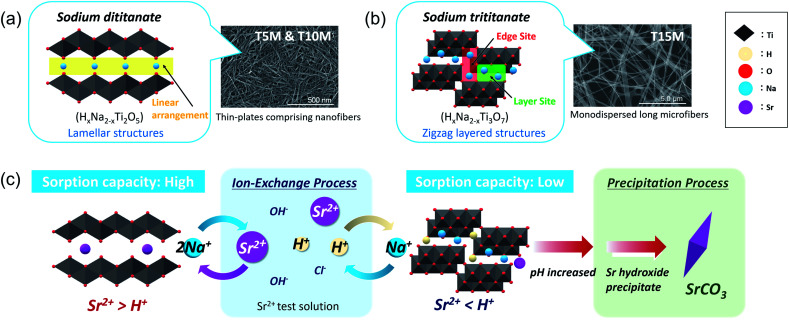
Schematic of the crystal structure of (a) dititanate (T5M and T10M) and (b) trititanate (T15M) phases and (c) their Sr^2+^ sorption behaviour.

The sodium titanate samples were classified into two groups: dititanate (H_*x*_Na_2−*x*_Ti_2_O_5_) and trititanate (H_*x*_Na_2−*x*_Ti_3_O_7_) phases (Scheme 1). The first group contained sodium dititanate (T5M and T10M), which has lamellar structures comprising the two-dimensional layers of edge and corner-sharing TiO_6_ octahedra, and cation (M^+^) interlayers.^26–28^ The SST, *i.e.*, T10M, was the dititanate phase, similar to that investigated in a report.^36^ In contrast, the sodium trititanate structure (T15M), as the second group, exhibited a zigzag layered structure comprising three units of TiO_6_ octahedra and an M^+^ interlayer.^14,27,32,43^ Na^+^ in the trititanate phase (T15M) existed at two different sites: edge sites and layer sites.^14,17,32^ Yang *et al.* reported that Na^+^ at edge sites can easily exchange ions compared with that of at layer sites, based on the substantial deformation of the (003) plane of sodium trititanate after sorption testing.^14,17,32^ In the present study, the same phenomenon of a decreasing 003 diffraction intensity of T15M was observed due to the strong interaction between Sr^2+^ and the titanate layer (Fig. S6d†),^14,32^ and therefore, it is assumed that the remaining Na was primarily located at “layer sites” in T15M. In contrast, Na^+^ in the interlayer of the dititanate structure was expected to be easily exchanged with cations because of the linear arrangement of its structure. In fact, in the present study, almost all of the Na^+^ ions in the interlayer of dititanate were exchanged with Sr^2+^ and H^+^*via* the ion-exchange reaction. The sorption selectivity of Sr^2+^ of sodium dititanate is higher than that of sodium trititanate. In the case of T15M, the solution pH rose to more than 10, and SrCO_3_ was precipitated despite the small amount of Na^+^ used for the ion-exchange reaction as compared with that of the dititanate cases (T5M and T10M). These results indicate that T15M exhibited a highly selective ion-exchange reaction of H^+^ relative to Sr^2+^ as compared with that of the dititanates. These selectivity differences are assumed to be due to the interlayer distance derived from the crystal structure and crystallinity. The ionic radius of Sr^2+^ (1.18 Å) is larger than that of H^+^ (−0.18 Å),^31^ and Sr^2+^ was proposed to be exchanged from the ionic state into sodium titanate as shown in the FT-EXAFS analysis. Therefore, Sr^2+^ would be difficult to intercalate into T15M, which has a narrow interlayer distance compared to that of T5M and T10M. In addition, the zigzag layered structure in the trititanate (T15M) with high crystallinity probably affects the ion-exchange reaction selectivity. Comparing T5M and T10M, which are the same dititanate phases, the crystallinity of T5M tended to be slightly higher than that of T10M, despite the irregular shape of T5M, because some large-size plate-crystals existed in T5M (Fig. S3b†). The Sr^2+^ sorption properties of T5M and T10M were almost the same, and thus, we suggest reducing the synthetic concentration of alkaline for SST formation, while considering the advantage of morphological uniformity.

Sodium dititanate is expected to be a promising water purification material for Sr^2+^ under weakly acidic conditions. Although sodium trititanate exhibited a low Sr^2+^ sorption capacity, the results of our study have demonstrated that the design of the crystal structure of sodium titanate can control the sorption mechanism and selectivity of the incorporated cation species. In the case of ion-exchange reactions, the water molecules contributes as cations, and the pH change and the effects of coexisting ions affect the reactions more complexly. The differences in the ionic radii, valency, hardness of the coexisting ions, and the solubility product of their precipitates are expected to affect the sorption properties of sodium titanate.^31,36^ Therefore, the effects of coexisting ions on the adsorption capacity of SST in wastewater need to be investigated further under model conditions. However, our basic findings will contribute to the development of SSTs as highly efficient purification materials for water treatment.

## Conclusions

To investigate the effective optimal-synthesis conditions for SST mats, layered sodium titanates were synthesised by an alkaline hydrothermal process using 5–15 M NaOH solutions. Layered sodium titanates were tested as water purification materials for Sr^2+^ removal. As-synthesised sodium titanates were classified into two groups, dititanate and trititanate with different crystal structures. The H^+^/Na^+^ ratio of dititanate was higher than that of trititanate, and dititanate exhibited a higher Sr^2+^ sorption capacity than trititanate. The Sr^2+^ sorption phenomenon was distinctive, as trititanate ion-exchanged and precipitated SrCO_3_, whereas dititanate experienced only ion-exchange. Na^+^ in trititanate exhibited a high selectivity for ion-exchange to H^+^ in the solution, and some of these Na^+^ ions remained after the reaction. As a result, trititanate had a low Sr^2+^ sorption capacity, despite the high content of Na^+^, and the primary Sr^2+^ removal mechanism was SrCO_3_ precipitation due to pH increase. In contrast, Na^+^ in dititanate exhibited highly selective ion-exchange with Sr^2+^ in solution, and this tendency suppressed the rapid rise in pH and self-precipitation. This difference was assumed to be due to the crystal structure and interlayer distance. Further improvement of the highly selective Sr^2+^ ion-exchange reaction of SSTs by optimising the crystal structure and composition may lead to the development of two-dimensional nanomaterials for water purification.

## Author contributions

TG and TS conceived and designed the present study. YK and TG performed the sample preparation, characterisation, and the data analysis for the work. YK wrote the drafts of the manuscript, and TG edited it. All authors discussed the result, commented on drafts of the manuscript, and approved the final report.

## Conflicts of interest

There are no conflicts to declare.

## Supplementary Material

RA-011-D1RA03088D-s001

## References

[cit1] Chu S., Majumdar A. (2012). Nature.

[cit2] McCollum D. L., Zhou W., Bertram C., De Boer H. S., Bosetti V., Busch S., Després J., Drouet L., Emmerling J., Fay M., Fricko O., Fujimori S., Gidden M., Harmsen M., Huppmann D., Iyer G., Krey V., Kriegler E., Nicolas C., Pachauri S., Parkinson S., Poblete-Cazenave M., Rafaj P., Rao N., Rozenberg J., Schmitz A., Schoepp W., Van Vuuren D., Riahi K. (2018). Nat. Energy.

[cit3] Dresselhaus M. S., Thomas I. L. (2001). Nature.

[cit4] Cao J., Cohen A., Hansen J., Lester R., Peterson P., Xu H. (2016). Science.

[cit5] Burns P. C., Ewing R. C., Navrotsky A. (2012). Science.

[cit6] Dyer A., Pillinger M., Harjula R., Amin S. (2000). J. Mater. Chem..

[cit7] Komarneni S., Roy R. (1982). Nature.

[cit8] Paulus W. J., Komarneni S., Roy R. (1992). Nature.

[cit9] Goto T., Cho S. H., Lee S. W., Sekino T. (2018). J. Ceram. Soc. Jpn..

[cit10] Lantzsch J., Bushaw B. A., Herrmann G., Kluge H.-J., Monz L., Niess S., Otten E. W., Schwalbach R., Schwarz M., Stenner J., Trautmann N., Walter K., Wendt K., Zimmer K. (1995). Angew. Chem., Int. Ed. Engl..

[cit11] Lüning K. G., Frölén H., Nelson A., Rönnbäck C. (1963). Nature.

[cit12] Pors Nielsen S. (2004). Bone.

[cit13] U.S. Environmental Protection Agency (2014). Announcement of Preliminary Regulatory Determinations for Contaminants on the Third Drinking Water Contaminant Candidate List (USEPA). Fed. Regist..

[cit14] Yang D. J., Zheng Z. F., Zhu H. Y., Liu H. W., Gao X. P. (2008). Adv. Mater..

[cit15] Ishikawa Y., Tsukimoto S., Nakayama K. S., Asao N. (2015). Nano Lett..

[cit16] Dhandole L. K., Chung H. S., Ryu J., Jang J. S. (2018). ACS Sustainable Chem. Eng..

[cit17] Yang D., Zheng Z., Liu H., Zhu H., Ke X., Xu Y., Wu D., Sun Y. (2008). J. Phys. Chem. C.

[cit18] El-Kamash A. M. (2008). J. Hazard. Mater..

[cit19] Wang K., Wang F., Chen F., Cui X., Wei Y., Shao L., Yu M. (2019). ACS Sustainable Chem. Eng..

[cit20] Handley-Sidhu S., Renshaw J. C., Moriyama S., Stolpe B., Mennan C., Bagheriasl S., Yong P., Stamboulis A., Paterson-Beedle M., Sasaki K., Pattrick R. A. D., Lead J. R., MacAskie L. E. (2011). Environ. Sci. Technol..

[cit21] Goto T., Sasaki K. (2016). Powder Technol..

[cit22] Sasaki K., Goto T. (2014). Ceram. Int..

[cit23] Sun Y., Wang X., Ding C., Cheng W., Chen C., Hayat T., Alsaedi A., Hu J., Wang X. (2016). ACS Sustainable Chem. Eng..

[cit24] Nahar M. S., Zhang J. (2013). ACS Sustainable Chem. Eng..

[cit25] Takahatake Y., Shibata A., Nomura K., Sato T. (2017). Minerals.

[cit26] Zhao B., Lin L., He D. (2013). J. Mater. Chem. A.

[cit27] Zhao B., Chen F., Jiao Y., Zhang J. (2010). J. Mater. Chem..

[cit28] Zhu H. Y., Lan Y., Gao X. P., Ringer S. P., Zheng Z. F., Song D. Y., Zhao J. C. (2005). J. Am. Chem. Soc..

[cit29] Zhang Y., Jiang Z., Huang J., Lim L. Y., Li W., Deng J., Gong D., Tang Y., Lai Y., Chen Z. (2015). RSC Adv..

[cit30] Yuan Z. Y., Su B. L. (2004). Colloids Surf., A.

[cit31] Li N., Zhang L., Chen Y., Fang M., Zhang J., Wang H. (2012). Adv. Funct. Mater..

[cit32] Yang D., Liu H., Zheng Z., Sarina S., Zhu H. (2013). Nanoscale.

[cit33] Sun X., Li Y. (2003). Chem.–Eur. J..

[cit34] Merceille A., Weinzaepfel E., Barré Y., Grandjean A. (2011). Adsorption.

[cit35] Kunishi H., Hagio T., Kanimoto Y., Ichino R. (2020). Sci. Adv. Mater..

[cit36] Kondo Y., Goto T., Sekino T. (2020). RSC Adv..

[cit37] Kuwahara Y., Fujie Y., Yamashita H. (2017). ChemCatChem.

[cit38] Izawa H., Kikkawa S., Koizumi M. (1982). J. Phys. Chem..

[cit39] Farges F., Brown G. E., Rehr J. J. (1997). Phys. Rev. B: Condens. Matter Mater. Phys..

[cit40] Britvin S. N., Lotnyk A., Kienle L., Krivovichev S. V., Depmeier W. (2011). J. Am. Chem. Soc..

[cit41] Thorpe C. L., Lloyd J. R., Law G. T. W., Burke I. T., Shaw S., Bryan N. D., Morris K. (2012). Chem. Geol..

[cit42] Carroll S. A., Roberts S. K., Criscenti L. J., Day P. A. O. (2008). Geochem. Trans..

[cit43] Yang D., Zheng Z., Yuan Y., Liu H., Waclawik E. R., Ke X., Xie M., Zhu H. (2010). Phys. Chem. Chem. Phys..

