# Supplementary material for: Sr2+ sorption property of seaweed-like sodium titanate mats: effects of crystallographic properties
Source: RSC Adv. 2021 May 24;11(30):18676–84. doi: 10.1039/d1ra03088d (PMC9033459; doi:10.1039/d1ra03088d)
Supplement: RA-011-D1RA03088D-s001 [file RA-011-D1RA03088D-s001.pdf]

Electronic Supplementary Material (ESI) for RSC Advances.
This journal is © The Royal Society of Chemistry 2021

Supporting Information

Sr^{2+} sorption property of seaweed-like sodium titanate mats: effects of crystallographic properties

Yoshifumi Kondo,[a,b] Tomoyo Goto,[*, a,c] and Tohru Sekino[*,a]

[a]The Institute of Scientific and Industrial Research (ISIR-SANKEN), Osaka University, 8-1 Mihogaoka, Ibaraki, Osaka 567-0047, Japan

[b]Division of Materials and Manufacturing Science, Graduate School of Engineering, Osaka University, 2-1 Yamadaoka, Suita, Osaka 565-0871, Japan

[c]Institute for Advanced Co-Creation Studies, Osaka University, 1-1 Yamadaoka, Suita, Osaka 565-0871, Japan

Table of contents

	Pages
**Materials**	**S3**
**Preparation and Sr sorption test of protonated titanates**	**S3**
**Figure S1 – S9**	**S4 – S12**
**Table S1 – S2**	**S13**

Materials

Titanium sulfate solution ($Ti(SO_4)_2$, 30 %), sodium hydroxide (NaOH, 97.0 %), titanium dioxide (anatase, 98.5 %), strontium chloride ($SrCl_2$, 95.0 %), and strontium carbonate ($SrCO_3$, 99.99 %) were purchased from FUJIFILM Wako Pure Chemical Corporation, Osaka, Japan. Sodium metatitanate ($Na_2Ti_3O_7$) was obtained from Sigma-Aldrich Co. LLC., St. Louis, United States. All chemicals were used as received without further purification.

Preparation and Sr sorption test of protonated titanates

To investigate the effects of H^+ on ion-exchange reaction of titanates, protonated titantes of T10M and T15M were prepared. T10M and T15M were well-washed using the 1M HCl shacking for 30 min. They were marked as T10M_HCl and T15M_HCl, respectively. After that, Sr sorption test was conducted by using the procedure described above.

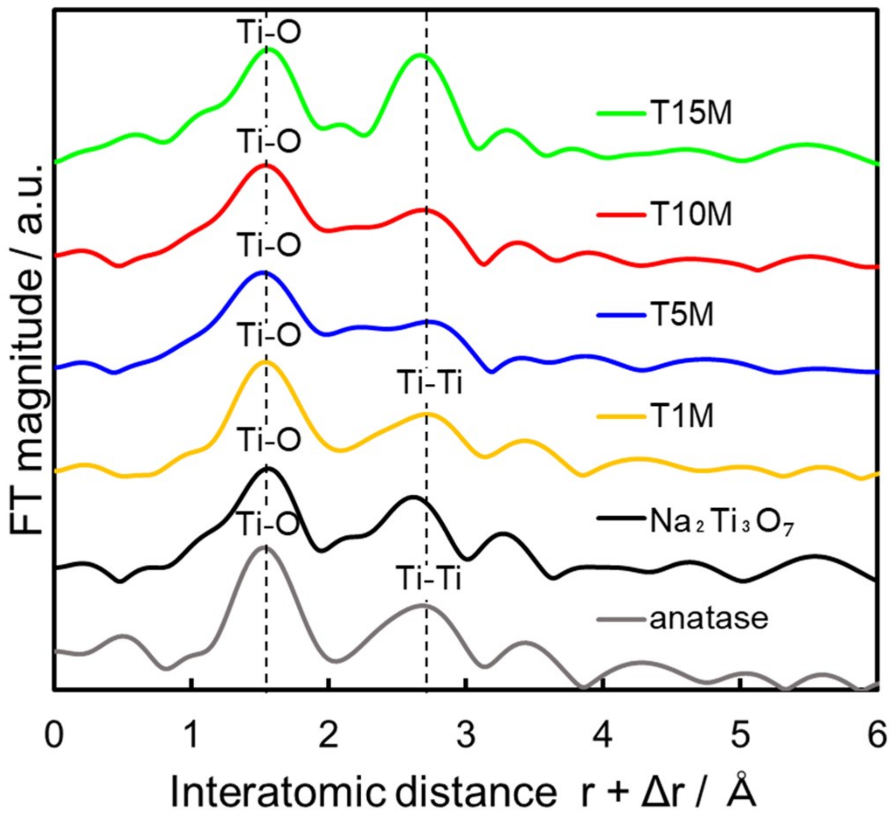

Fig. S1 Ti K-edge FT-EXAFS spectra of the reference samples (anatase and $Na_2Ti_3O_7$) and TXM samples.

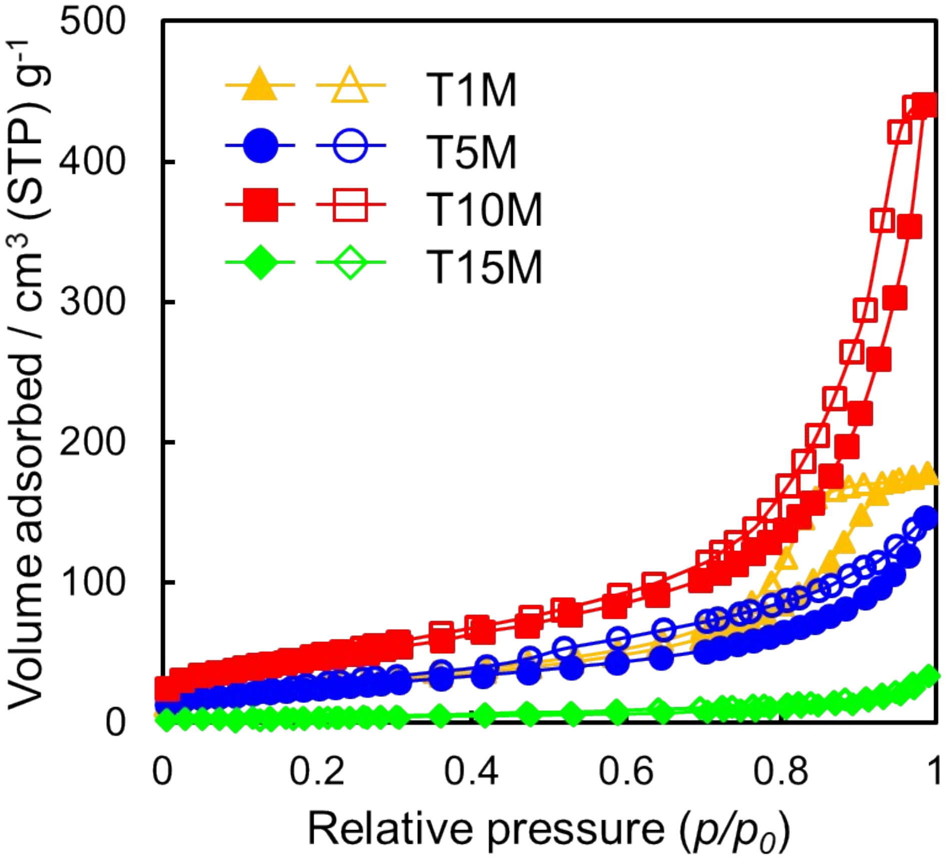

Fig. S2 N_2 adsorption-desorption isotherms of TXM sample.

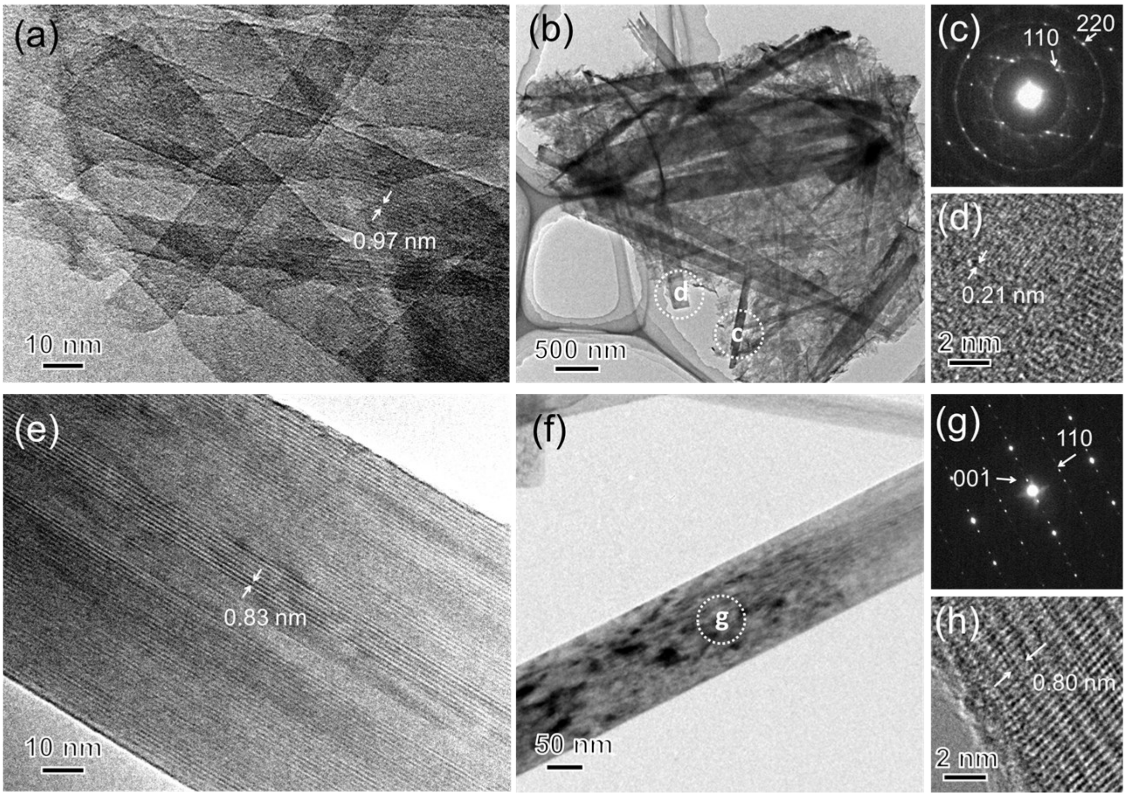

Fig. S3 TEM images, SAED pattern, and HRTEM images of the (a-d) T5M and (e-h) T15M samples.

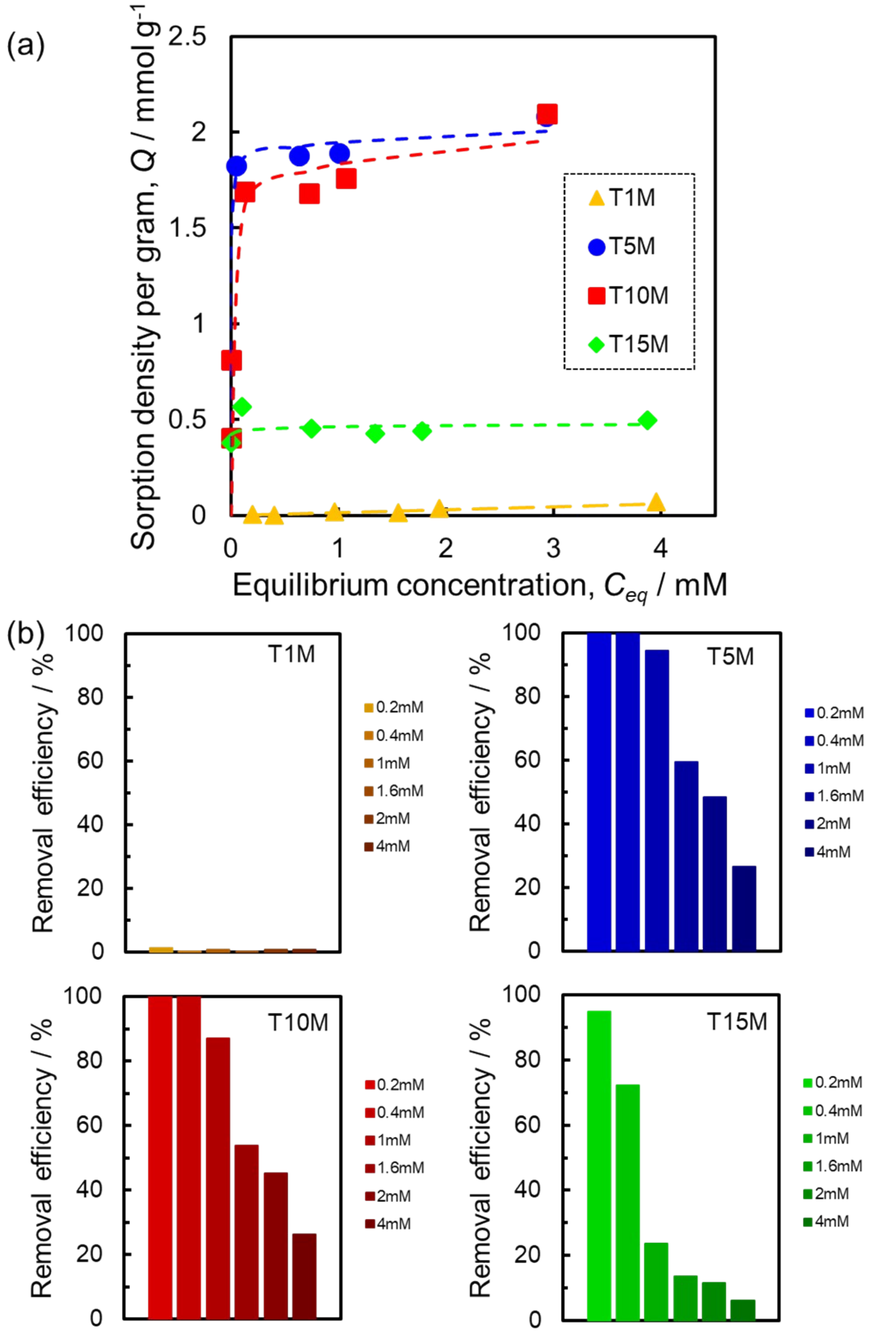

Fig. S4 (a) Sr^{2+} sorption isotherms fitted with the Freundlich plot of TXM samples, and (b) removal efficiency of Sr^{2+} regarding initial concentration of each TXM sample.

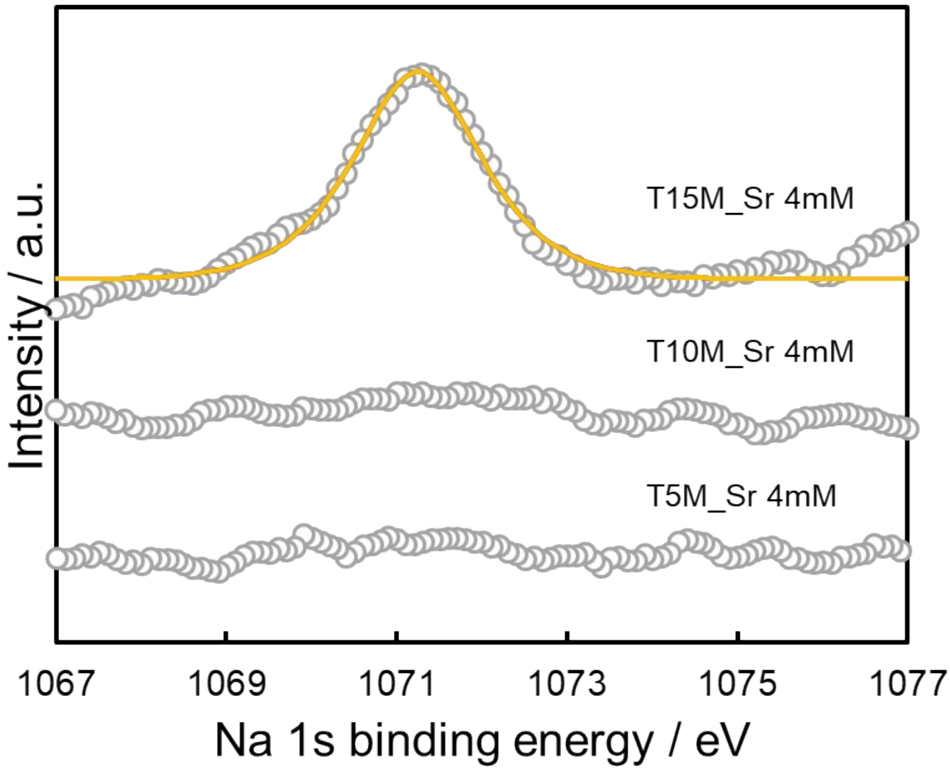

Fig. S5 Na 1s XPS spectra of T5M, T10M and T15M after the sorption test using 4 mM $SrCl_2$ solution.

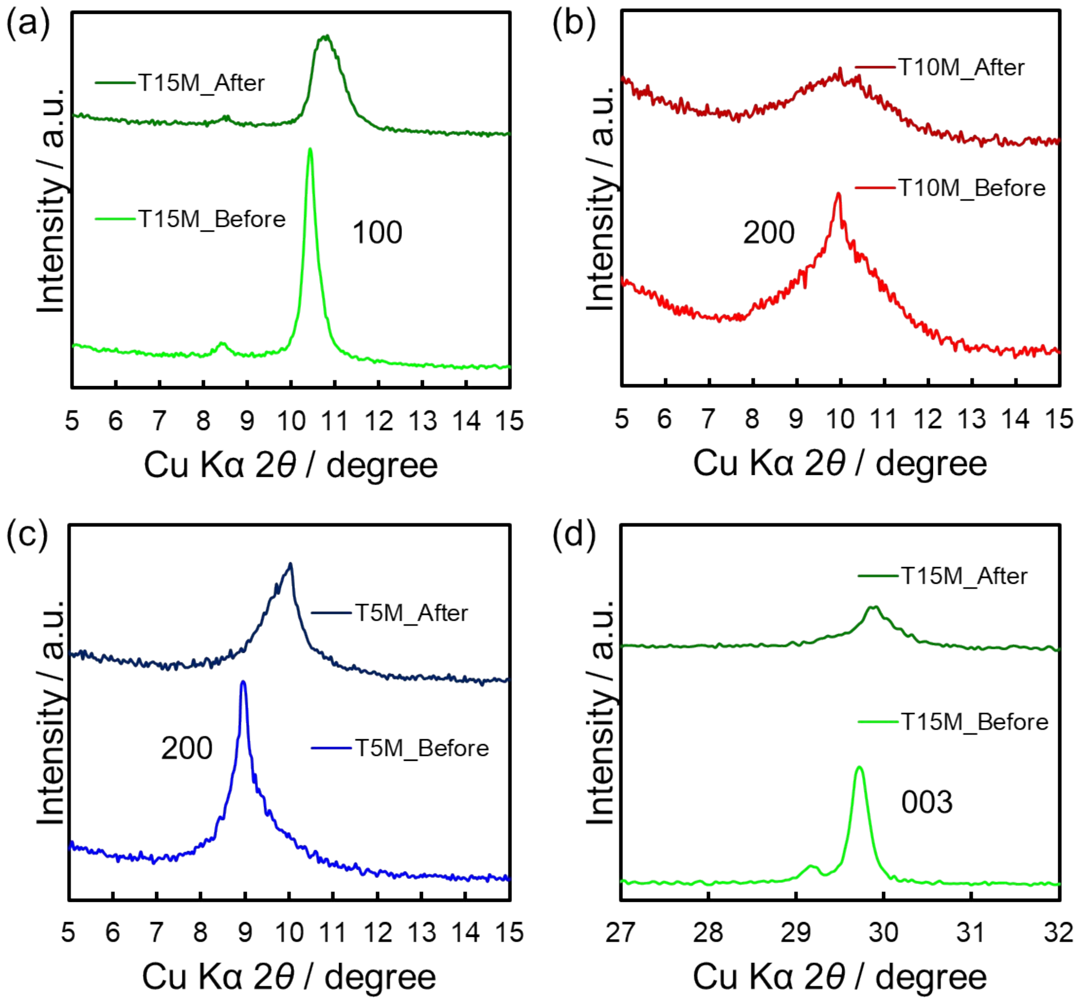

Fig. S6 Enlarged XRD patterns of TXM samples before and after the sorption test using 4 mM $SrCl_2$ solution: (a) T15M in the range of 5° to 15°, (b) T10M in the range of 5° to 15°, (c) T5M in the range of 5° to 15°, and (d) T15M in the range of 27° to 32°.

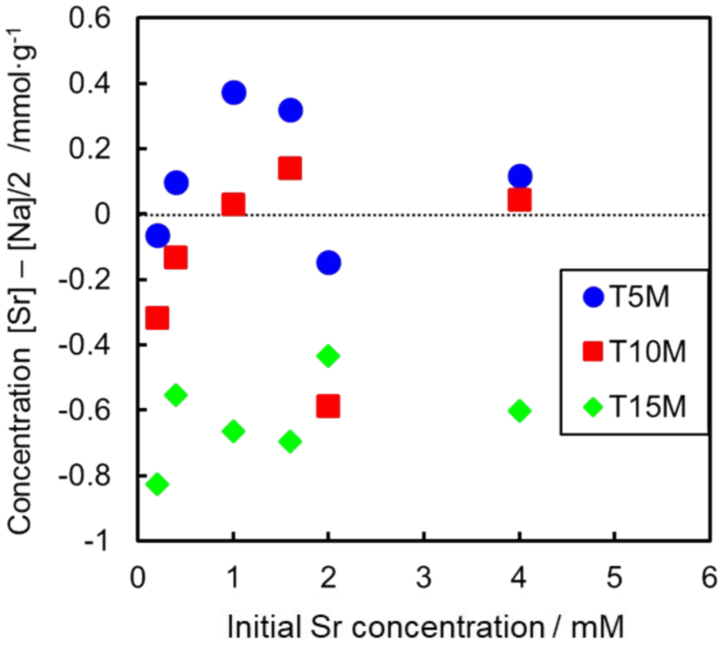

Fig. S7 Changes in the concentration of subtracted sorption [Sr] from released [Na]/2 against the initial Sr concentration after the sorption test.

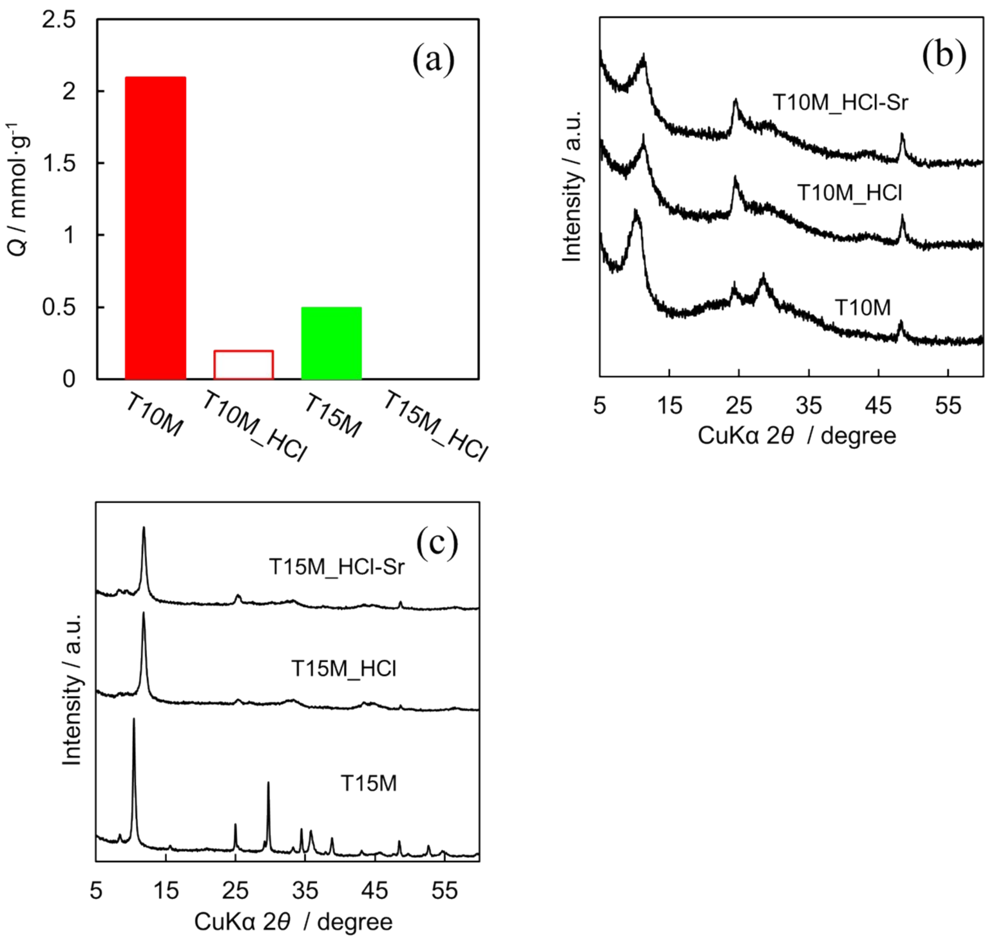

Fig. S8 (a) Sorption density of Sr on samples with or without HCl washing, and XRD pattern of (b) T10M and (c) T15M samples before and after Sr sorption test.

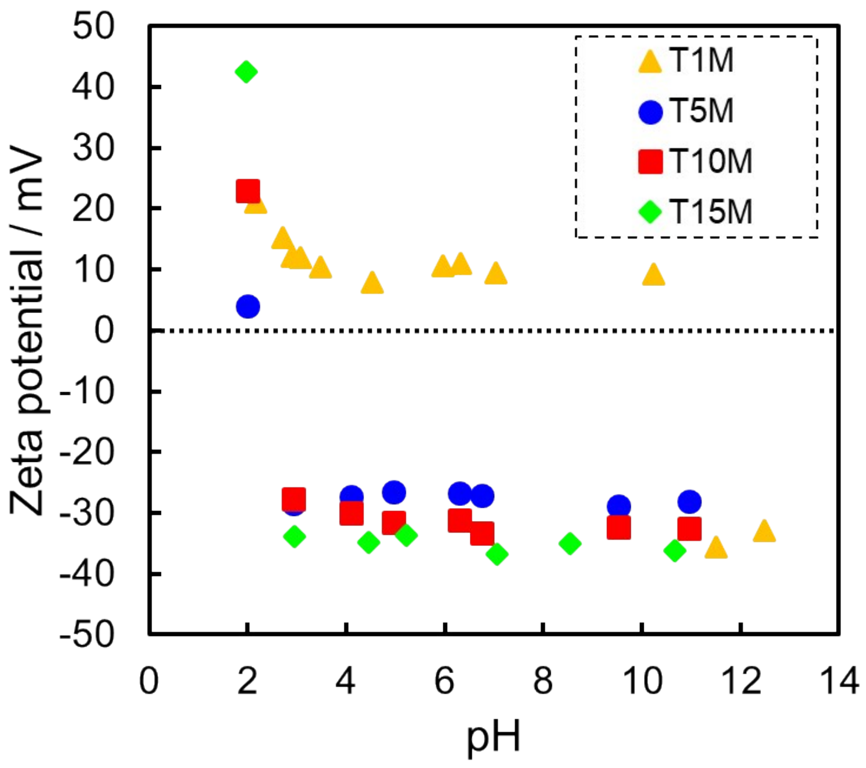

Fig. S9 pH dependence of zeta potential of TXM samples. The pH of test solution was adjusted to 2-13 by additions of HCl and NaOH solutions.

Table S1 Langmuir isotherm and Freundlich isotherm constants for Sr^{2+} sorption on TXM Samples.

Sample	Langmuir			Freundlich		
	Q_{max} (mmol/g)	K (L/mmol)	r^2	K_F (mmol/g(L/mmol)$^{1/n}$)	$1/n$	r^2
T1M	-0.08	-0.12	0.09	0.01	0.98	0.77
T5M	2.08	34.58	1.00	1.95	0.03	0.67
T10M	2.09	14.99	0.99	1.83	0.06	0.59
T15M	0.49	14.01	0.99	0.46	0.02	0.10

Table S2 The value of d_{200} of T5M and T10M, and d_{100} of T15M samples before and after Sr^{2+} sorption test using 4 mM $SrCl_2$ solution.

Sample	d_{h00} before[a] (Å)	d_{h00} after[a] (Å)
T5M	9.87	8.93
T10M	8.88	8.87
T15M	8.40	8.34

[a] Calculated by the Bragg equation.
